# Esterase, Glutathione S-Transferase and NADPH-Cytochrome P450 Reductase Activity Evaluation in *Cacopsylla pyri* L. (Hemiptera: Psyllidae) Individual Adults

**DOI:** 10.3390/insects12040329

**Published:** 2021-04-07

**Authors:** Dolors Bosch-Serra, Marcela A. Rodríguez, Jesús Avilla, María José Sarasúa, Xavier Miarnau

**Affiliations:** 1 Department of Sustainable Plant Protection, Food and Agriculture Research Institute (IRTA), ETSEA Campus, Av. Rovira Roure 191, 25198 Lleida, Spain; 2 Departamento de Zoología, Facultad de Ciencias Naturales y Oceanográficas, Universidad de Concepción, Casilla 160-C, Concepción 4030000, Chile; marcerodriguez@udec.cl; 3 Department of Crop and Forest Sciences, Agrotecnio-CERCA Center, University of Lleida (UdL), Av. Rovira Roure 191, 25198 Lleida, Spain; jesus.avilla@udl.cat (J.A.); ambly.ander@gmail.com (M.J.S.); 4 Fruit Production Program, Food and Agriculture Research Institute (IRTA), Fruitcentre, Parc Científic i Tecnològic Agroalimentari de Lleida (PCiTAL), Parc de Gardeny, Edifici Fruitcentre, 25003 Lleida, Spain; xavier.miarnau@irta.cat

**Keywords:** *Cacopsylla pyri*, insecticide resistance, metabolic resistance, enzymatic activity, individual analysis

## Abstract

**Simple Summary:**

The resistance of pest insects to insecticides is a growing problem in all types of crops. *Cacopsylla pyri* (L.) (Hemiptera: Psyllidae) is a key pest of pear orchards in Spain, and the large number of insecticide treatments necessary for its control may contribute to the emergence of resistance. Laboratory insecticide toxicity (mortality assays) and biochemical assays (to know the enzymatic mechanisms of the insects in acquiring resistance) are necessary to confirm the existence of this resistance. All the previous methodologies developed to evaluate enzyme activity in *C. pyri* to date have been performed using “pools” of adults. In this study, we determined the optimal working conditions for the evaluation of the enzymatic activities in individual insects. Determining the frequency of resistant individuals within a population could be used as an indicator for the evolution of insecticide resistance over time. Two laboratory strains, one of them selected with cypermethrin, and two field populations were analyzed for this purpose. The data obtained revealed that one of the three resistance mechanisms studied had a high level of activity and was present in a high proportion of insects in the population selected with the insecticide as well as and in the field populations. These results validated the applied methodology.

**Abstract:**

*Cacopsylla pyri* (L.) (Hemiptera: Psyllidae) is a key pest of pear orchards in Spain. The large number of insecticide treatments necessary for control may be an important contributor to the emergence of resistance. Laboratory toxicity and biochemical assays are necessary to validate the existence of insecticide resistance and establish the underlying mechanisms. All the methodologies developed to evaluate enzyme activity in *C. pyri* to date have incorporated “pools” of adults to detect minimum activity ranges. In this study, we determined the optimal working conditions for evaluation of the activities of esterase, glutathione S-transferase and NADPH-cytochrome P450 reductase in individual insects via colorimetric methods using a microplate reader. The main factors affecting enzymatic analysis activity, such as enzyme source and substrate concentration, filter wavelength, buffer pH, reaction time and additives, were evaluated for optimization. Determining the frequency of resistant individuals within a population could be used as an indicator for the evolution of insecticide resistance over time. Two laboratory strains, one of them selected with cypermethrin, and two field populations were analyzed for this purpose. The data obtained revealed high values and great variation in the activity ranges of esterase (EST) in the insecticide-selected population as well as in the field populations validating the applied methodology.

## 1. Introduction

*Cacopsylla pyri* (L.) (Hemiptera: Psyllidae) is the principal pest of pear orchards in Spain. The species is distributed throughout the European continent, along with parts of Asia and Tunisia [1]. The main damage induced by this pest is indirect. Honeydew excreted by psylla larvae during the feeding process can cause spots and necrosis in fruits, with subsequent commercial downgrading [2,3]. Moreover, sooty mold commonly develops on honeydew on all parts of the tree. Other indirect damage in Spain and the Mediterranean region transmitted by *C. pyri* includes the diseases pear decline and peach yellow leaf roll caused by the phytoplasma “*Candidatus* Phytoplasma pyri” [4]. Both diseases involve yellowing, the progressive loss of tree vigor and production and, in some cases, the collapse and death of trees [5,6,7].

In the fruit-growing area of Lleida (northeast Spain) comprising 47% of the Spanish pear surface crop [8], pesticide control is the most commonly used technique for the elimination of pear psylla [9,10]. However, pesticide control of this pest is difficult due to several biological factors, such as overlapping generations, high reproduction potential and the presence of honeydew, which acts as a larval protector [2,11], in addition to the reduction of authorized insecticides and the development of resistance to several insecticides [12,13,14]. Resistance to cypermethrin and low susceptibility to amitraz and abamectin have additionally been reported in some Spanish populations collected in this area [15,16].

The mechanisms generally involved in insecticide resistance include metabolic resistance through the upregulation or alteration of the catalytic properties of detoxifying enzymes and insensitivity at the action target site through changes in receptors or enzymes targeted by insecticides [17,18,19,20,21,22]. The activity of detoxification enzymes, such as esterase (EST), glutathione S-transferase (GST) and cytochrome P450 polysubstrate monooxygenase (PSMO), can be effectively detected and quantified using biochemical techniques, such as electrophoresis, colorimetry or immunoserology [23]. 

Gomori [24] proposed a method for measuring EST activity based on the ability to hydrolyze aryl esters. This methodology was introduced into entomological research by Van Asperen [25] and adapted for use with microplate readers [26,27,28]. Alpha and β-naphthyl acetates are the most commonly used substrates to quantify EST activity in *C. pyri* and *Cacopsylla pyricola* [29,30,31], as well as other insects [32,33,34].

The ability of certain glutathione S-transferases to combine with specific substrates (1,2-dichloro-4-nitrobenzene, 1-chloro-2,4-dinitrobenzene (CDNB) and ρ-nitro benzyl chloride, among others) in the presence of glutathione has been applied for enzymatic analysis of GST activity in numerous insects. For the evaluation of GST in *C. pyri* and *C. pyricola* [29,30,31] and other insects [32,34,35], CDNB is the most commonly used substrate owing to the highly specific activity of most transferases [36,37]. 

To determine PSMO activity, substrates, such as benzo(α)pyrene, metoxiresorufin, ethoxyresorufin, cytochrome C and ethoxycoumarin, have been successfully used in several insecticide-resistant populations [23,38]. However, in *C. pyri*, PSMO activity has only been measured using methodologies involving 7-ethoxycoumarin O-deethylation (ECOD) [31,39]. Nicotinamide adenine dinucleotide phosphate (NADPH)-cytochrome P450 reductase (CPR) is a key enzyme in the cytochrome P450 system. It is a membrane-bound protein that transfers electrons to P450s in the metabolization and detoxification of substrates and xenobiotics [40,41]. The expression level of CPR has been associated with insect susceptibility to the insecticide and has been also used to measure the P450 activity in psyllids [42] as well as other insects [43].

The involvement of detoxification enzymes in *C. pyri* insecticide resistance was first reported by Berrada et al. [31] based on studies involving synergists and biochemical assays. The group showed that increased PSMO activity, together with the presence of acetylcholinesterase (AChE), was responsible for the high level of resistance to an organophosphate insecticide (monocrotophos) in a selected laboratory population. In *C. pyricola*, Van de Baan and Croft [30] demonstrated the association of resistance to azinphos-methyl and fenvalerate with the increased activity of EST and PSMO detoxification enzymes in adult summer forms and azinphos-methyl-induced reduction of penetration through cuticles in adult winter forms. 

Limited studies to date have focused on biochemical analyses in *C. pyri* [13,31,39,44]. Moreover, all earlier experiments have been conducted using “pools” of adults to ensure minimum activity measurement, with no consideration of the sex. Using pools can mask the presence of individuals with high levels of enzyme activity when these individuals are present at a low frequency. Individual analysis may facilitate the determination of inter-individual variability and more robust statistical analysis by including more replicates in each population [34]. Knowledge of the frequency of resistant individuals in a population is important to establish the rate of resistance development (resistant-susceptible composition) and enzymatic activity ranges as well as the influence of migration on the evolution of resistance [45,46].

The objectives of this study were to optimize the measurements of the enzymatic activities in single *C. pyri* adults. Therefore, efficient colorimetric techniques and optimal conditions for measuring EST, GST and CPR enzyme activities were established. To validate these techniques, populations with different responses of toxicity to insecticides were needed to ensure a possible variability in their enzymatic activity. Thus, toxicity bioassays with cypermethrin were performed in four populations: two field populations collected in the Ebro Valley area and two laboratory strains, one selected with increasing concentrations of cypermethrin and the other strain reared with no insecticide selection. Mean enzymatic activity and frequency distribution of enzymatic activities of the insects of these four populations, taking into account the sex of the individual insects, were calculated following the new methodology.

## 2. Materials and Methods

### 2.1. Insects

Winterform *C. pyri* field adults were selected for enzyme assays and were obtained with “beating trays” and sucked with a pooter device.

To obtain optimal enzymatic activity measurements, it would be appropriate to work with both resistant and susceptible populations [25,28,47]. Thus, two field populations designated “Fraga” and “Aitona1”, collected during winter 2005–2006, that had previously shown differences in susceptibility to multiple insecticides [15] were tested. In addition, two laboratory *C. pyri* strains were studied, both collected in the same orchard during 2005 and reared during 14 generations in the laboratory. The laboratory strain named “PoalS” was reared without any insecticide pressure and the “PoalRCyp” strain was continuously selected with increasing concentrations of cypermethrin that induced approximately 60% mortality in adults (0.050 g a.i./L to 0.200 g a.i./L).

Detailed information on field orchards is not provided in this report, since our main objective was to optimize the methodology and not compare insecticide susceptibility of the orchard pest populations. Samples of each population, depending on what they will be used for, were collected and stored at the fridge or at −70 °C until experimental use [13,31].

### 2.2. Chemicals

A commercial formulation of the pyrethroid cypermethrin was used for toxicity bioassays (10%; Afrisec, Lainco, Barcelona, Spain). Reagents for enzymatic bioassays, specifically, phenylmethylsulfonyl fluoride (PMSF), dithiothreitol (DTT), reduced glutathione (GSH), Triton X−100, cytochrome C, β-nicotinamide adenine dinucleotide phosphate (NADP), D-glucose-6-phosphate, glucose-6-phosphate dehydrogenase, Fast Garnet (diazonium ion), α-naphthyl acetate (α-NA) and α-naphthol, were purchased from Sigma-Aldrich (St Louis, MO, USA). Ethylenediaminetetraacetic acid disodium salt dehydrate (EDTA), monosodium phosphate dihydrate and sodium dodecyl sulfate (SDS) were obtained from J. T. Baker (Center Valley, PA, USA); 1-chloro-2,4-dinitrobenzene (CDNB) from Fluka (St Louis, MO, USA) and disodium phosphate dihydrate were obtained from Merck (Madrid, Spain).

### 2.3. Insecticide Bioassays

The adults were treated by topical application following the methodology used by Berrada et al. [48] and Buès et al. [49], among others. The adults, previously anesthetized with CO_2_, received a 0.5 µL drop of the insecticidal solutions prepared with a 40% aqueous alcoholic solution, without insecticide in the case of the controls, using a Hamilton microsyringe. After the treatment, they were maintained in a glass tube (1.2 cm in diameter and 7.5 cm high) and were not fed. Mortality was recorded 24 h after the treatment. Six to eight insecticide concentrations were tested. For each dose, a minimum of four repetitions of 10 individuals were treated. Each test included an aqueous alcoholic solution control of equal size.

### 2.4. Optimization of the Biochemical Methodology Adapted for Use with a Microplate Reader

#### 2.4.1. Preliminary Tests 

Before standardizing the general methodology, various factors were analyzed, such as absorbance filter wavelength, substrate concentration, enzymatic concentration of the crude homogenate samples, buffer pH, enzyme reaction time and dye concentration. Each factor was analyzed within the range of optimal conditions while maintaining practicability.

Due to their interaction, and in order to carry out the optimization tests, it was necessary to set some values necessary for the calculation of enzymatic activities in EST, GST and CPR: twenty µL of enzymatic extract (1 adult homogenized in 250 µL phosphate buffer), phosphate buffer pH 7, α-NA 1 mM, with an incubation time of 15 min at 30 °C, Fast Garnet 10 mM and a post-incubation time of 15 min at ambient temperature (~23 °C) for EST; 20 µL enzymatic extract (1 adult homogenized in 150 µL phosphate buffer), phosphate buffer pH 7, GSH 5 mM with a pre-incubation time of 15 min at 30 °C, CDNB 30 mM, EDTA (1 mM) and DTT (0.1 mM) in homogenization and reaction buffers for GST; and 20 µL enzymatic extract (1 adult homogenized in 80 µL phosphate buffer), phosphate buffer pH 9, NADPH 1x, with a pre-incubation time of 15 min at 30 °C, cytochrome C 1 mM, EDTA (1 mM) and DTT (0.1 mM) in homogenization and reaction buffers and Triton X−100 (0.5%) in homogenization buffer for CPR. Absorbance rates during incubation times were measured for GST and CPR every minute for 30 min. 

Variable numbers of adults were used for each test (specified in every section) but with an equal number of males and females in all cases. Two absorbance readings per adult were taken, with each test repeated at different times (2–4). Controls (with no enzyme source) were used to consider natural substrate degradation.

##### Filter Wavelength

To achieve maximum absorbance readings, it was necessary to select an appropriate filter wavelength for the microplate reader. A continuous reading spectrometer (UNICAM UV/VIS Spectrometer UV2, Cambridge, UK) with a 1-cm cuvette light path was employed.

The recommended wavelengths for EST activity measurements vary among different reports in psylla species (Civolani et al. [44] (450 nm) and Berrada et al. [31] (527 nm) for *C. pyri*; Van de Baan and Croft [30] (600 nm) for *Psylla pyricola* Foerster and other insect genera [32,33,35,50]. Accordingly, our tests were performed using a wavelength range of 450 and 650 nm. Measurements were conducted by adding α-naphthol (1 mM) as a standard product or an enzyme source for the hydrolysis of α-NA to produce α-naphthol. The assay conditions were as follows: 20 mL enzyme extract (one adult homogenized in 180 mL phosphate buffer), phosphate buffer pH 7, incubation for 15 min at 30 °C, Fast Garner 10 mM, and post-incubation for 15 min at room temperature (~23 °C).

The wavelength of 340 nm used in the measurement of GST activity [36] has been successfully employed for enzyme analysis in different insects [30,31,32,33,43,51]. For CPR activity measurement, a wavelength of 550 nm was proposed by Masters et al. [52], which was successfully applied for corresponding enzymatic analyses in other insect populations [44]. Accordingly, 340 and 550 nm were used as wavelengths for GST and CPR measurements, respectively, in our study.

##### Substrate Concentration

We examined a range of substrate concentrations to determine those suitable for different enzyme activity measurements, i.e., 0.1–8.0 mM for α-NA (EST activity), 1–30 mM for CDNB (GST activity) and 0.1–2.0 mM for cytochrome C (CPR activity). In each assay, 6–7 concentrations were tested with 4–8 repetitions. The Michaelis–Menten constant (*K*_m_) was calculated to ensure saturating substrate concentrations [53].

##### Enzyme Source Concentration

Twenty µL of different crude homogenate concentrations as the enzymatic source were analyzed, along with different volumes of homogenization buffer (40–500 µL per adult) equivalent to between 0.5 and 0.04 adults per well, with 4–6 repetitions per test. The concentrations ranged between a minimum below which manipulation errors can occur, considered three times the standard error in the activity of the control [34], and a maximum above which could lead to a potential lack of substrate [53]. 

##### Reaction Buffer pH 

The influence of reaction buffer (pH 5–10) in enzyme activity measurements was analyzed due to its influence on substrate–enzyme binding and enzyme catalytic activity [54,55]. The occurrence of non-enzymatic reactions should be minimal in controls [36]. The number of repetitions varied from 3 to 13 depending on the substrate.

##### Enzymatic and Non-Enzymatic Reaction Times

Several assays were performed to analyze the influence of time in the different reactions in the plate, enzymatic (reactions between the substrate and the enzymatic source) and non-enzymatic (the color reaction or enzyme extract with additives reaction). The tested times ranged between 1 and 60 min and the number of repetitions between 2 and 16, depending on the reaction.

#### 2.4.2. Enzyme Source Preparation

Whole adults were used to prepare the crude homogenates as enzyme sources. Each adult was sexed and homogenized in an amount of sodium phosphate buffer (50 mM, pH 7 [56]) depending on the optimal enzyme source concentration needed. Different additives were assayed in preliminary tests to improve enzyme extraction according to the bibliography, including the addition of Triton X-100 (0.5%) to the extraction buffer and EDTA (1 mM) + DTT (0.1 mM) and PMSF (0.4 mM) to both homogenization and reaction buffers [29,30,31]. 

Homogenization was performed in an Eppendorf tube using a manual homogenizer at approximately 4 °C. Extracts were centrifuged at 10,000× *g* for 10 min at 4 °C (MPW-350R, Irmeco, Bielsko-Biała, Poland) to separate the supernatant [31]. The supernatant sample from each individual was collected and frozen at −20 °C until analysis.

#### 2.4.3. General Procedure for Measurement of Enzyme Activity 

Activities of three enzyme groups related to chemical detoxification (EST, GST and CPR) were analyzed with colorimetric methods using specific substrates for each enzyme. All enzyme activity measurements were conducted at 30 °C [54] based on changes in absorbance using the VICTOR3™ Multilabel microplate reader (PerkinElmer Life and Analytical Sciences, Madrid, Spain). 

##### Esterase (EST) Activity 

The endpoint assay reported by Van Asperen [25] for houseflies and modified for *C. pyri* [31] was optimized and adapted for use with microplate readers. Assays were performed by adding 20 µL enzyme source per well on a 96-well transparent microplate (Greiner Bio-One, Madrid, Spain) with 160 µL sodium phosphate buffer (50 mM at a given pH) and 20 µL substrate solution (α-NA) diluted in distilled water (with 1% acetone). In previous assays, a range of enzyme source and substrate concentrations were tested to optimize measurement conditions. The mixture was incubated at 30 °C [54] for a specific time period (1 to 60 min). The enzymatic reaction was terminated post-incubation at room temperature by adding 20 µL of solution containing SDS (35 mg/mL) and Fast Garnet in distilled water. This new reaction between naphthol and diazonium ion produced a colored product, measured based on absorbance in the visible spectral range. Five different concentrations of Fast Garnet (ranging between 2.5 and 40 mM) were assayed to evaluate its influence on EST activity, calculated as nmol hydrolyzed substrate·min^−1^·mg^−1^ protein using α-naphthol as standard.

To obtain the α-naphthol standard curve, α-NA was replaced by α-naphthol and the enzyme source by homogenization buffer. Different concentrations of α-naphthol (ranging between 0.02 and 1.00 mM) with several replicates were prepared to generate linear regression of absorbance values versus concentration of α-naphthol. 

##### Glutathione S-Transferase (GST) Activity 

The proposed methodology for the measurement of GST activity was based on reports by Habig et al. [36] and Habig and Jakoby [37]. An aliquot (20 µL) of enzyme source was added to each transparent microplate well with 170 µL GSH in sodium phosphate buffer solution (50 mM at a given pH) as a cofactor for substrate conjugation. The mixture was pre-incubated at 30 °C from 1 to 60 min to standardize reagent temperature. The reaction was initiated by adding 10 µL CDNB solution in ethanol. The GST-catalyzed substrate conjugation formed a colored product. To evaluate product formation, the absorbance rate (the increase in absorbance) was read at 25–30 °C [37] every minute during the 30-min incubation period. Specific enzyme activity was expressed as nmol substrate conjugated·min^−1^·mg^−1^ protein using a CDNB molar extinction coefficient of 9.6 mM^−1^·cm^−1^ [36]. A range of CDNB as well as GSH concentrations (0.5–20 mM) was assayed to determine the optimal reaction conditions.

##### NADPH–Cytochrome P450 Reductase Enzyme Activity (CPR)

The methodology for analysis of CPR activity was proposed by Masters et al. [52] and modified by Ortego et al. [43]. However, this protocol has not been applied for *C. pyri* adults to date. 

The assay commenced with the introduction of a 20-µL enzyme extract into each well, with 160 µL sodium phosphate buffer (50 mM at a given pH) solution containing an NADPH-generating system as a cofactor necessary to conjugate the substrate. NADPH 1× comprised a solution of NADP (0.6 mM), 2.8 mM D-glucose-6-phosphate and 0.28 units of glucose-6-phosphate dehydrogenase. We examined a range of concentrations (1× to 0.25×) to establish the optimum solution for use. After pre-incubation of the sample at 30 °C for 0 to 30 min, the reaction commenced with the addition of 20 µL cytochrome C solution in sodium phosphate buffer (50 mM, pH 7), leading to a reduction in the substrate and consequent color changes. Various concentrations of cytochrome C were examined for optimization of the assay. The absorbance rate was read every minute over the 30-min incubation period. CPR activity was expressed as nmol substrate reduced·min^−1^·mg^−1^ of protein, using the molar extinction coefficient for the reduced form of cytochrome C of 27.6 mM^−1^·cm^−1^ [57].

### 2.5. Protein Concentration Measurement

Protein assessment in enzyme sources was performed using the method described by Bradford [58] with the Pierce BCA Protein Assay Kit (Thermo Scientific and Life Science Research Products, Rockford, IL, USA) using bovine serum albumin (BSA) as standard. 

### 2.6. Enzyme Activity Evaluation in Populations of C. pyri

Adult winterforms collected from the two orchards were processed following the methodology described above according to the results obtained in optimization assays. Twenty adults comprising equal numbers of males and females were analyzed for each population and substrate. Aliquots of diluted individual homogenates were added in duplicates to individual wells of a 96-well microtiter plate for immediate analysis. 

Specific enzyme activity was expressed as nmol substrate hydrolyzed, conjugated or reduced min^−1^·mg^−1^ of protein.

### 2.7. Statistical Analysis

A probit analysis using the program POLO Plus [59] was performed, and the LC_50_, the LC_90_ and their 95% fiducial limits were calculated. Two LC_50_ were considered significantly different when their fiducial limits did not overlap [59]. The resistant ratio (RR) relative to the most susceptible population was calculated.

The absorbance, product formation rate, and EST, GST and CPR enzymatic activities of field adults were analyzed via ANOVA followed by a Tukey–Kramer Test (α = 0.05) with JMP 14.2.0 (SAS Institute Inc, 2018). Absorbance and product formation rate variables were transformed through “log (x + 1)” to satisfy normality and homogeneity data conditions analyzed using Kolmogorov–Smirnov and Levene tests, respectively. The associations between several factors and absorbance and reaction rates were analyzed using linear and hyperbolic regression with SAS Enterprise Guide 7.1 (2014) and TableCurve^TM^ (1996) software programs, respectively.

## 3. Results and Discussion

### 3.1. Insecticide Bioassay Results

The results of the probit analysis and the RR of the tested populations are shown in Table 1. The most susceptible population to cypermethrin was PoalS, the population reared in the laboratory without any insecticide pressure. The selected strain PoalRCyp showed a significantly lower susceptibility than the laboratory population PoalS, with an RR of 7.5, but was not significantly different from the field population Aitona1. The other field population tested, Fraga, showed the same susceptibility as PoalS and was significantly more susceptible than the other two populations. Therefore, among these populations, clear differences in the toxicity related to the insecticide existed and were appropriate to test the new enzymatic analysis methodology.

### 3.2. Preliminary Tests for Enzyme Activity Measurement 

#### 3.2.1. Filter Wavelength

**Figure 1:**
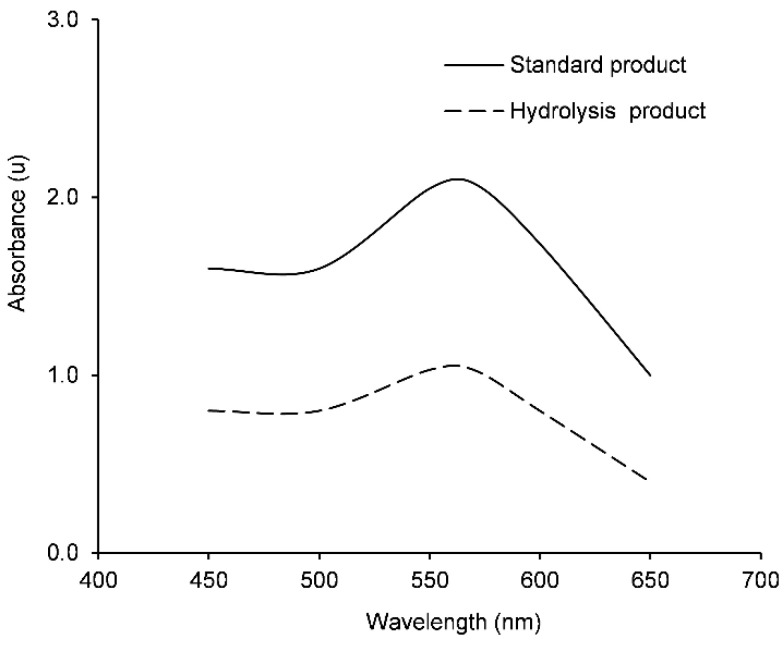
Absorbance spectrum of reaction products (α-naphthol + Fast Garnet). Alpha-naphthol was obtained from the standard product (1 mM) or enzyme source via hydrolysis of α-naphthyl acetate (1 mM). Absorbance measurements were conducted in a continuous reading spectrometer with a 1-cm light path cuvette (*n* = 3).

Absorbance values obtained in EST activity measurements with the standard product, α-naphthol, and those obtained via hydrolysis of the enzymatic source showed similar patterns (Figure 1). Values obtained with the standard product were higher than those with the hydrolysis reaction since the amount of naphthol produced by hydrolysis is lower than that added directly as a standard product. The optimal working wavelength range to obtain maximum absorbance was between 550 and 580 nm (maximum, 570 nm). Absorbance curve patterns related to wavelength were similar to those obtained by Dary et al. [28] and fitted with wavelengths applied by Van de Baan and Croft [30] and Berrada et al. [31]. However, studies on *Cacopsylla permixta* Burckhardt and Hodkinson by Esmaeily et al. [60] and *C. pyri* by Civolani et al. [44] measured absorbance at 450 nm for EST activity. Based on the collective results and available filters, 550 nm was selected as the wavelength for experimental use in our study.

For GST and CPR activity measurements, recommended wavelengths of 340 and 550 nm were selected, respectively, based on good preliminary results.

#### 3.2.2. Substrate Concentration

The recommended substrate concentration varies according to published studies, from values close to *K*_M_ [36] to higher values close to the saturation point (7–10 times *K*_M_) [34,53,54].

**Figure 2:**
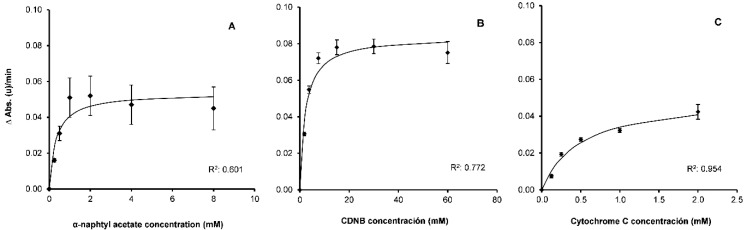
Substrate concentration (α-naphthyl acetate (**A**), 1-chloro-2,4-dinitrobenzene (CDNB) (**B**) and cytochrome C (**C**)) influence the reaction rate (ΔAbs.(u)/min). The continuous line in the figures indicates a theoretical hyperbolic curve obtained by fitting the Michaelis–Menten equation to the data. The error bars correspond to standard error (total number of insects tested, A: *n* = 28, B: *n* = 56, C: *n* = 12).

In Figure 2, the hydrolysis rate as the function of substrate concentration is presented as the reaction rate. Values higher than 1 mM α-NA did not increase the reaction rate of EST activity (Figure 2A). In addition, higher concentrations of substrate led to a lack of solubility. Upon adjusting the data obtained in the tests to a hyperbolic curve [54], the *K*_M_ value obtained was 0.30 (*p* < 0.001). The selected α-NA concentration for the assay was 1 mM, which was higher than *K*_M_ but below the saturation concentration. This dose was distinct from that used by Civolani et al. [44] (100 mM) and Berrada et al. [31] (0.1 mM) with *C. pyri* and Esmaeily et al. [60] (30 mM) with *C. permixta*.

For GST activity measurements, CDNB concentrations above 15 mM did not lead to increased reaction rates (Figure 2B). The calculated *K*_M_ value was 2.25 (*p* < 0.001). In keeping with previous criteria, the selected concentration for the assay was 15 mM, similar to that recommended by Habig et al. [36]. Civolani et al. [43] used a concentration of 0.4 mM with *C. pyri,* while Esmaeily et al. [60] used 63 mM. 

For CPR activity measurements (Figure 2C), cytochrome C concentrations above 2 mM were difficult to use due to low solubility. In addition, high cytochrome C concentrations produced very high absorbance rate values in the controls, supporting spontaneous non-enzymatic degradation of the substrate. The calculated *K*_M_ was 0.50 (*p* < 0.001) and selected concentration was 1 mM.

#### 3.2.3. Enzyme Source Concentration and Use of Cofactors 

**Figure 3:**
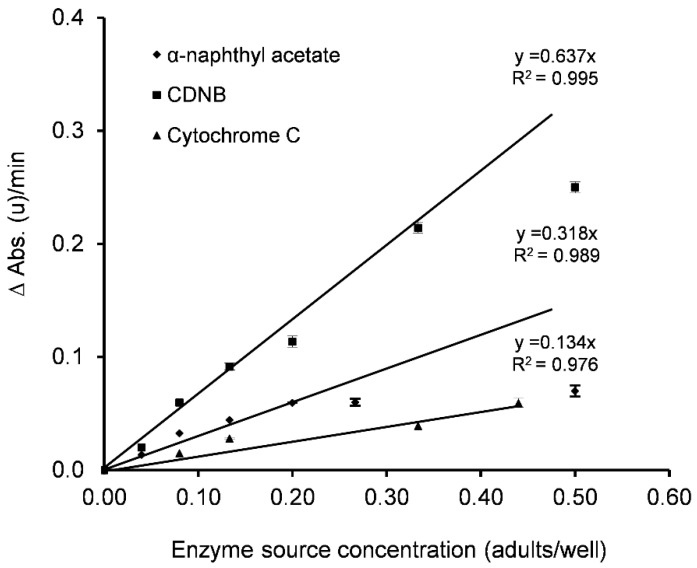
Influence of enzyme source concentration on reaction rates (ΔAbs.(u)/min) with the three respective substrates (α-naphthyl acetate, CDNB and cytochrome C). Error bars correspond to standard error (total number of insects tested, α-naphthyl acetate: *n* = 56, CDNB: *n* = 28, cytochrome C: *n* = 24).

The reaction rate obtained for α-NA and CDNB showed a linear relationship with enzyme source concentrations between 0–0.2 (R^2^ = 0.989) and 0–0.33 (R^2^ = 0.995) adults per well, respectively (Figure 3). In terms of α-NA activity, the maximum reaction rate should never be reached (0.09 ΔAbs.(u)/min according to the naphthol calibration line (R^2^ = 1.000)), since all the naphthyl acetate would be converted to naphthol by EST, potentially leading to a lack of substrate. For GST activity, the minimum required absorbance increments were 0.05 ΔAbs.(u)/min [36]. An intermediate concentration that would allow working with field populations with higher or lower activity than those in the tests was selected, specifically, 0.08 adults per well for EST and 0.13 adults per well for GST. 

The reaction rate for cytochrome C had a linear relationship with enzyme source concentrations between 0 and 0.44 adults per well (R^2^ = 0.976) (Figure 3). The use of lower concentrations could induce errors. Accordingly, the optimum value was selected as 0.25 adults per well. PSMO activity studies in *C. pyri* and *C. pyricola* have been previously performed using microsomal preparations (30,31), as these samples provide a high concentration of P-450 enzymes [61]. Civolani et al. [44] used homogenates of *C. pyri* nymphs with sixfold amounts of extract supernatant for PSMO calculation (16.0 nymphs) than for EST and GST (2.67 nymphs), while Esmaeily et al. [60] performed studies on adults of *C. permixta* homogenates using the equivalent of 16.7, 30.0 and 6.7 adults for PSMO, GST and EST analyses, respectively. 

Previous enzymatic assays to determine psyllid insecticide resistance were performed using “pools” of more than 50 individuals [31,44,60]. Experiments from the current study have highlighted the possibility of analyzing insecticide resistance mechanisms at the individual level and even mechanisms in the same individual. 

The concentrations of two necessary cofactors, GSH and NADPH-generating systems, for GST and CPR activity measurements, respectively, were also determined in preliminary trials. The optimal values were 5 mM for GSH (*p* < 0.001) and NADPH 1× (*p* < 0.001), similar to those proposed by Habig et al. [36] and Masters et al. [52], respectively.

#### 3.2.4. Reaction Buffer pH

**Figure 4:**
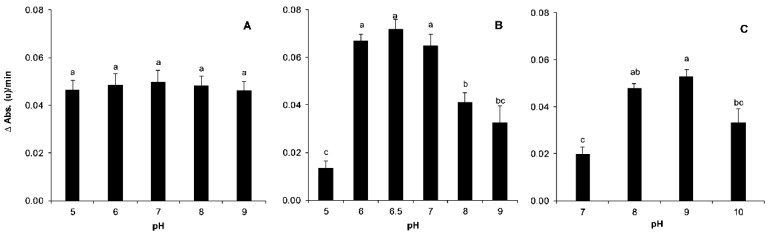
Influence of pH on reaction rates (ΔAbs.(u)/min) with the three respective substrates (α-naphthyl acetate (**A**), CDNB (**B**) and cytochrome C (**C**)). The error bars correspond to standard error (total number of insects tested, (**A**) *n* = 65, (**B**): *n* = 75, (**C**): *n* = 12). Vertical bars with different letters are significantly different according to mean separation using Tukey–Kramer’s test (*p* < 0.05).

Absorbance was not significantly affected with different sodium phosphate buffer pH values for EST activity (*p* = 0.974; Figure 4A). Previously, Van Asperen [25] showed that activity was slightly dependent on pH according to the type of esterase. The pH value was selected as 7 to avoid enzyme degradation problems and improve substrate stability in the aqueous solution [25,28]. However, significant pH-dependent differences in the GST reaction rate were observed (*p* < 0.001; Figure 4B). The optimal pH in our working conditions was 6–7, with no significant differences among the values within this range. The selected value was 6.5, which was in the same range as that used by other authors [30,31,36,60].

The optimal pH conditions for reduction of cytochrome C were between 8 and 9, with no marked differences at these two values. However, differences were significant out of this range (*p* = 0.001; Figure 4C). The selected value for phosphate buffer was 9, similar to previous reports with other insects [43,52,62]. In this case, buffer had to be prepared immediately prior to enzymatic activity measurement, since the structures of some reagents could vary under high alkaline conditions.

#### 3.2.5. Reaction Time

**Figure 5:**
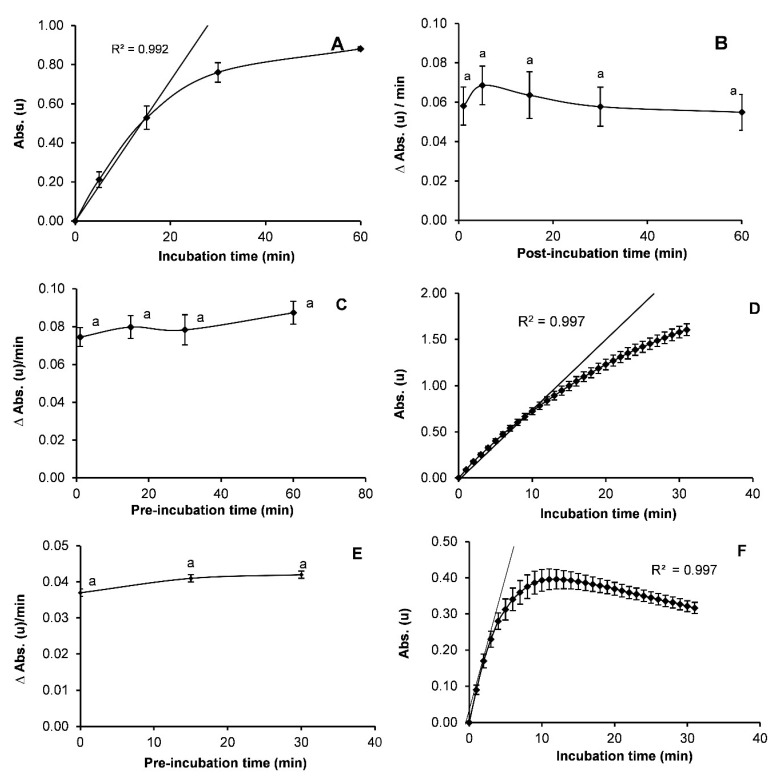
Influence of pre-incubation, incubation and post-incubation times on absorbance or absorbance rate (ΔAbs.(u)/min) with α-naphthyl acetate (**A**,**B**), CDNB (**C**,**D**) and cytochrome C (**E**,**F**) substrates. The error bars correspond to standard error (Total number of insects tested, (**A**) *n* = 8, (**B**) *n* = 20, (**C**) *n* = 32, (**D**) *n* = 20, (**E**) *n* = 6 and (**F**) *n* = 16). Absorbance rate values with different letters (**B**, **C** and **E**) are significantly different according to mean separation using Tukey-Kramer’s test (*p* < 0.05).

Absorbance during the incubation time of EST increased following a linear relationship (R^2^ = 0.996) between 0 and 15 min (Figure 5A). At higher time-points, absorbance began to decline and did not show a proportional increase. According to Dary et al. [28], the optimal time was between 10 and 30 min. Consistently, Berrada et al. [31] reported an optimal time of 20 min. Thus, 15 min was selected as the optimal incubation time for our assays. Regarding optimal post-incubation time, for the non-enzymatic reaction, no significant differences were observed between periods of 1 and 60 min (*p* = 0.391) (Figure 5B). To standardize all trials and ensure sufficient handling time, a period of 15 min was selected.

No significant differences in GST were observed among the pre-incubation times tested (*p* = 0.521; Figure 5C). Based on these results, 15 min was selected as the handling time to unify the temperature of all reagents. During the incubation period with readings taken every minute, times longer than 8 min did not present a linear relationship (Figure 5D; R^2^ = 0.997). Consequently, 5 min was selected as the time to calculate average product formation. 

For CPR activity, no significant differences in reaction rates were observed over the range of pre-incubation times tested (*p* = 0.05; Figure 5E). A time period of 15 min was subsequently selected to standardize the temperature for all tests. On the other hand, incubation times longer than 3 min did not present a linear relationship with absorbance (Figure 5F), and therefore, a reaction time of 2 min with measurements every minute was selected (R^2^ = 0.999).

#### 3.2.6. Dye Concentration

Significant differences in reaction rate as a function of Fast Garnet concentration were observed in EST activity measurement (*p* < 0.001). The reaction rate was markedly lower at 40 mM than at concentrations between 2.5 and 20 mM. Notably, the solution changed from liquid to a paste at concentrations above 20 mM, which could influence measurements. Therefore, 10 mM was selected to provide excess reagent to ensure that all the naphthol produced could react with the dye.

### 3.3. Additives for Enzyme Source Preparation

Additives are used to minimize enzyme denaturalization and degradation during their extraction and thus improve the measurement of enzyme activity [63]. Several trials with different additives were conducted (Table 2). No significant differences in EST activity were evident upon addition of Triton X-100 or EDTA + DTT (*p* = 0.958 and *p* = 0.231). However, PMSF induced a marked decrease in the reaction rate (*p* < 0.001). Triton X-100 was the detergent used for improving the enzyme extraction process and enhancing detection of EST in a *Culex quinquefasciatus* L. (Diptera: Culicidae) population resistant to organophosphates by Dary et al. [28], in *C. permixta* by Esmaeily et al. [60], and in *C. pyri* by Civolani et al. [44]. Berrada et al. [31] used EDTA to improve the EST activity measurement procedure.

In GST activity trials, the addition of EDTA + DTT to both homogenization and reaction buffers induced a significant increase in the reaction rate (*p* < 0.001) while PMSF had no effect (*p* = 0.099) and Triton X-100 triggered a significant decrease in the reaction rate (*p* = 0.008). Berrada et al. [31] and Civolani et al. [44] used Triton X-100 in the homogenization step to obtain the enzyme source for GST activity while Esmaeily et al. [60] used no additives. 

The reaction rate of CPR was improved by adding Triton X-100 (*p* = 0.028) in the homogenization process and EDTA + DTT (*p* < 0.001) in both buffers, while PMSF did not alter the enzymatic activity measurement (*p* = 0.258).

Accordingly, we decided to use no additives for EST activity measurements and include EDTA (1 mM) and DTT (0.1 mM) in homogenization and reaction buffers to improve the detection of GST activity. For CPR, Triton X-100 (0.5%) and EDTA + DTT were added to the homogenization buffer and EDTA + DTT to the reaction buffer.

### 3.4. Enzymatic Activity in Single Adults

Mean enzymatic activities of EST, GST and CPR from single individuals of the four *C. pyri* populations obtained following the optimized and standardized methodology are displayed in Table 3.

The insecticide-selected population PoalRCyp had significantly higher EST and CPR activity than PoalS. Aitona1 and Fraga, the field-collected populations, did not present significant differences in EST activity with PoalRCyp, selected with cypermethrin, but had significantly lower GST and CPR activity than both laboratory populations. 

**Figure 6:**
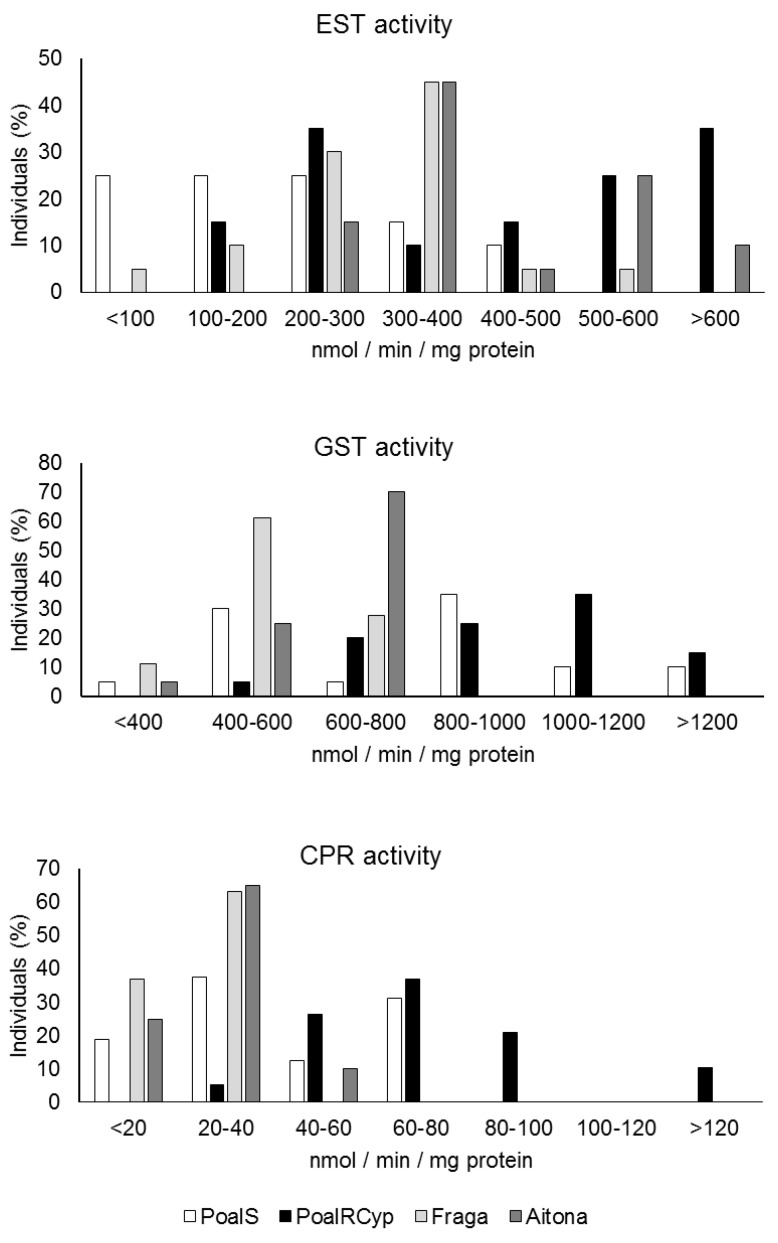
Frequency distribution of EST, GST and CPR activities (nmol of substrate ·min^−1^·mg^−1^ of protein) in psylla adults obtained from two laboratory-reared strains and two field populations collected in the Ebro Valley area (Spain). (Total number of insects tested, EST: *n* = 80; GST: *n* = 78; CPR: *n* = 74; *n* = 16–20 individuals per population).

Figure 6 depicts the frequency distribution of enzyme activities. The ranges in which the enzymatic activity of ESTs and CPRs is distributed in the two laboratory populations are notably different. 

The majority of PoalRCyp adults (85%) showed EST activity between the ranges 200–300 and >600 nmol·min^−1^·mg^−1^ of protein, while for PoalS, 75% of the values are between the ranges <100 and 200–300 nmol·min^−1^·mg^−1^ of protein. CPR activity was mainly distributed between the ranges <20 and 60–80 nmol·min^−1^·mg^−1^ of protein in PoalS, and between 40–60 and 80–100 nmol·min^−1^·mg^−1^ of protein in PoalRCyp, with 10% of adults presenting >120 nmol·min^−1^·mg^−1^ of protein activity values. These findings imply that these two enzymatic mechanisms seem to be involved in the detoxification of the pyrethroid cypermethrin, and, most importantly for our objective, that the applied methodology allows us to detect the enzymatic activity differences in single pear psylla adults. In the field populations, the range in which the EST activity values are distributed is very similar to the selected laboratory population PoalRCyp, especially for Aitona1. Civolani et al. [44] showed significantly higher EST activity in one field population of *C. pyri,* while Berrada et al. [31] found that PSMO was involved in the detoxification of monocrotophos. Nevertheless, to identify which detoxification mechanisms are present in the field, toxicity and enzymatic bioassays of different populations to different active components are essential. In addition, there are different ways to evaluate enzymatic activity [41], and different resistance mechanisms may be involved in the field populations as different mutations at the target side of insecticides. 

In general, there were no significant differences between sexes in any enzymatic activity. The sex of *C. pyri* adults has never been taken into account in enzyme activity measurements to date [13,30,31,44]. In other species, mainly Lepidoptera, conflicting results in this regard have been reported [64,65,66].

## 4. Conclusions

The biochemical methodology to determine resistance mechanisms to insecticides is unique for every insect and enzyme group, and it is therefore important to establish the appropriate protocols. In previous studies performed on *C. pyri* and *C. pyricola*, enzymatic assays were performed with pools of individual insects to detect minimum activity. In the current study, the methodology to evaluate activities of three enzymes, namely EST, GST and PSMO, was optimized and standardized for detection of activity in single individual adults. This individual analysis facilitated the determination of inter-individual variability and the frequency of resistant individuals within a population, which serves as an effective indicator of the evolution of resistance over time and highlights the emergence of possible combinations of different resistance mechanisms in an individual. The adults analyzed from the two laboratory strains, one of which was selected with cypermethrin (RR = 7.5), showed greater differences in the mean activity and in the distribution range of EST absorbance frequencies, indicating that these enzymes can be involved in the detoxification of the pyrethroid cypermethrin. The two analyzed field populations also presented mean and frequencies distribution of EST activity similar to the insecticide selected strain. The study, including insecticide toxicity and enzymatic bioassays, of multiple *C. pyri* populations of the Ebro Valley area is needed to determine the presence and extension of resistant insecticide populations and the implied mechanisms. In the case of detecting resistance to insecticides in field populations, in addition to the detoxification mechanisms examined in this article, other resistance mechanisms should be studied. This knowledge is important to implement effective anti-resistance strategies and establish baseline data before or soon after the introduction of new insecticides to the market. Simultaneously, our methodology enables analysis of larger populations, due to the small number of individuals required, and can aid in more robust statistical analysis owing to the examination of more replicates in each population. 

## Figures and Tables

**Table 1:** Probit analysis results and resistance ratios to cypermethrin obtained after topical applications on adults of *Cacopsylla pyri* obtained from two reared laboratory strains and from two field populations collected in the Ebro Valley area (Spain).

		Probit Analysis Parameters						
Population	N ^1^	Slope ± SE	LC_50_^2^	CI 95% ^3^	LC_90_	CI 95% ^3^	HF ^4^	RR ^5^
PoalS ^6^	230	1.32 ± 0.19	0.056 a	0.034–0.083	0.524	0.321–1.140	0.70	1.0
PoalRCyp ^7^	240	1.60 ± 0.33	0.422 b	0.248–0.655	2.677	1.475–9.072	0.53	7.5
Aitona1	320	2.55 ± 0.42	0.316 b	0.246–0.398	1.004	0.721–1.790	0.96	5.6
Fraga	320	2.61 ± 0.43	0.084 a	0.057–0.111	0.262	0.197–0.400	0.78	1.5

^1^ N = number of individuals tested. ^2^ Values of the lethal concentrations (LC) are g a.i./L. LC_50_ followed by the same letter are not significantly different (LC_50_ are considered significantly different when their CI do not overlap). ^3^ CI 95% = 95% confidence intervals. ^4^ HF = Heterogeneity factor. ^5^ RR = Resistance ratio calculated by dividing the LC_50_ of the strain tested by the LC_50_ of the laboratory susceptible strain. ^6^ Population reared in laboratory during 14 generations without any pesticide pressure. ^7^ Population reared in laboratory during 14 generations selected with increasing concentrations of cypermethrin.

**Table 2:** Influence of additives on reaction rate in esterase (EST), glutathione S-transferase (GST) and cytochrome P450 reductase (CPR) activity measurements.

Enzyme Group		Triton X-100 ^1^ (∆Abs.(u)/min ± SE)			EDTA + DTT ^2^ (∆Abs.(u)/min ± SE)			PMSF ^3^ (∆Abs.(u)/min ± SE)	
	n ^5^	Without	With	n ^5^	Without	With	n ^5^	Without	With
**EST ^4^**	16	0.058 ± 0.002 a	0.057 ± 0.002 a	40	0.051 ± 0.003 a	0.047 ± 0.003 a	48	0.056 ± 0.002 a	0.034 ± 0.003 b
**GST ^4^**	12	0.087 ± 0.010 a	0.053 ± 0.003 b	78	0.065 ± 0.003 b	0.094 ± 0.004 a	32	0.047 ± 0.005 a	0.057 ± 0.005 a
**CPR ^4^**	8	0.023 ± 0.003 b	0.038 ± 0.005 a	8	0.008 ± 0.001 b	0.020 ± 0.001 a	8	0.042 ± 0.008 a	0.030 ± 0.006 a

^1^ Triton X-100 (0.5%) in homogenization buffer. ^2^ Ethylenediaminetetraacetic acid disodium salt dehydrate (EDTA) (1 mM) + dithiothreitol (DTT) (0.1 mM) in homogenization and reaction buffers. ^3^ Phenylmethylsulfonyl fluoride (PMSF) (0.4 mM) in homogenization buffer. ^4^ Values with different letters for the same activity and additive are significantly different according to mean separation using Tukey–Kramer’s test (*p* < 0.05). ^5^ *n* = total number of adults evaluated in each trial.

**Table 3:** Enzymatic activities of EST, GST and CPR in adults of *C. pyri* from two reared laboratory strains and two field populations collected in the Ebro Valley area (Spain). *N =* number of insects tested.

		**EST Activity ± SE (n) ^1^**	
**Populations**	**Total**	**Male**	**Female**
PoalS ^4^	193.61 ± 28.65 (20) b	168.00 ± 44.02 (10) A	219.22 ± 37.20 (10) A
PoalRCyp ^5^	346.77 ± 34.40 (20) a	336.08 ± 54.71 (10) A	357.46 ± 44.47 (10) A
Aitona1	438.39 ± 42.05 (20) a	496.04 ± 71.40 (10) A	380.72 ± 40.36 (10) A
Fraga	303.52 ± 22.68 (20) a	298.66 ± 30.81 (10) A	308.39 ± 34.90 (10) A
		**GST activity ± SE (n) ^2^**	
PoalS ^4^	837.99 ± 65.02 (20) a	714.07 ± 91.24 (10) B	961.91 ± 78.19 (10) A
PoalRCyp ^5^	937.23 ± 50.90 (20) a	849.11 ± 74.25 (10) A	1025.35 ± 60.81 (10) A
Aitona1	625.59 ± 34.13 (20) b	668.29 ± 35.51 (10) A	582.88 ± 57.02 (10) A
Fraga	533.44 ± 21.82 (18) b	510.24 ± 24.02 (10) A	562.45 ± 34.14 (8) A
		**CPR activity ± SE (n) ^3^**	
PoalS ^4^	40.39 ± 5.03 (16) b	40.45 ± 7.88 (8) A	40.32 ± 6.82 (8) A
PoalRCyp ^5^	77.52 ± 7.26 (19) a	82.93 ± 11.58 (9) A	72.66 ± 9.34 (10) A
Aitona1	25.99 ± 1.87 (20) c	32.49 ± 2.05 (10) A	19.49 ± 1.10 (10) B
Fraga	23.12 ± 1.52 (19) c	23.13 ± 1.74 (10) A	23.12 ± 2.68 (9) A

^1^ Activity measured in nmol of hydrolyzed substrate mg protein^−1^ min^−1^; ^2^ activity measured in nmol of conjugated substrate mg protein^−1^ min^−1^; ^3^ activity measured in nmol of reduced substrate mg protein^−1^ min^−1^; ^4^ population reared in laboratory during 14 generations without no pesticide pressure; ^5^ population reared in laboratory during 14 generations selected with increasing concentrations of cypermethrin. Different lowercase letters indicate significant differences among the populations in the same enzymatic activity. Different capital letters indicate significant differences between sexes.

## Data Availability

Data are contained within the article.

## References

[1] CAB Internacional Home Page Distribution Maps of Plant Pests Map 671: Cacopsylla pyri 2005.

[2] Atger P., Atger P. (1982). La biologie du psylle du Poirier. Le Psylle du Poirier.

[3] García de Otazo J., Sió J., Torà R., Torà M. (1992). Peral: Control Integrado de Plagas y Enfermedades.

[4] Seemüller E., Schneider B. (2004). Taxonomic description of “Candidatus Phytoplasma mali” sp. nov., “Candidatus Phyto-plasma pyri” sp. nov., and “Candidatus Phytoplasma prunorum” sp. nov., the casual agents of apple proliferation, pear decline and European stone fruit yellows, respectively. Int. J. Syst. Evol. Microbiol..

[5] Lemoine J., Simon M.C., Costard F., Bossu V. (1998). Le dépérissement du poirier ou “Pear Decline”. Phytoma Défense Végétaux.

[6] Garcia-Chapa M., Sabaté J., Lavina A., Batlle A. (2005). Role of Cacopsylla pyri in the epidemiology of pear decline in Spain. Eur. J. Plant Pathol..

[7] Sabaté J., Rodon J., Artigues M., Laviña A., Batlle A. (2018). Transmission of ‘Candidatus Phytoplasma pyri’ by naturally infectedCacopsylla pyrito peach, an approach to the epidemiology of peach yellow leaf roll (PYLR) in Spain. Plant Pathol..

[8] MAPA (Ministerio de Medio Ambiente y Medio Rural) (2017). Anuario de Estadística Agroalimentaria 2017.

[9] Avilla J., Artigues M., Martí S., Sarasúa M.J. (1992). Parasitoides de Cacopsylla pyri (L.) (=Psylla pyri (L.)) presentes en una plantación comercial de peral en Lleida no sometida a tratamientos insecticidas. Bol. San. Veg. Plagas.

[10] Avilla J., Miarnau X., Rodríguez M., Bosch D., Artigues M., Sarasúa M.J. (2005). Resistencia de Cydia pomonella (L.) (Lepidoptera: Tortricidae) y de Cacopsylla pyri (L.) (Hemiptera: Psyllidae) a insecticidas. Phytoma España.

[11] Stäubli A., Hächler M., Pasquier D., Antonin P., Mittaz C. (1992). Dix années d’experiences et d’observations sur le psylle commun du poirier Cacopsylla (=Psylla) pyri L. en Suisse romande. Rev. Suisse Vitic. Arboric. Hortic..

[12] Stäubli A. (1983). Moyens de lutte biologiques et biotechniques contre les insectes. Rev. Suisse Vitic. Arboric. Hortic..

[13] Buès R., Toubon J.F., Rieux R., d’Arcier F. (1994). Polymorphisme des estérases et résistance aux insecticides chez un homoptère Cacopsylla pyri (L.). C. R. L’Acad. Sci. Sér. III Sci. Vie..

[14] Buès R., Toubon J.F., Boudinhon L. (1996). Le psylle du poirier. Résultats préliminaires sur la résistance aux insecticides en France. Phytoma Déf. Vég..

[15] Miarnau X., Artigues M., Sarasúa M.J. (2007). Evaluation of pear psylla, Cacopsylla pyri (L.) susceptibility to cypermethrin in pear orchards of Lleida, Spain. IOBC WPRS Bull..

[16] Miarnau X., Artigues M., Sarasúa M.J. (2010). Susceptibility to abamectin of pear psylla Cacopsylla pyri L. (Hemiptera: Psyllidae) in pear orchards of north-east Spain. IOBC WPRS Bull..

[17] Oppenoorth F.J., Kerkut G.A., Gilbert L.C. (1985). Biochemistry and genetics of insecticide resistance. Comprehensive Insect Physiology, Biochemistry and Pharmacology.

[18] Brown T.M., Brogdon W.G. (1987). Improved Detection of Insecticide Resistance Through Conventional and Molecular Techniques. Annu. Rev. Entomol..

[19] Georghiou G. (1994). Principles of insecticide resistance management. Phytoprotection.

[20] Scott J.A. (1995). The molecular genetics of resistance: Resistance as a response to stress. Fla. Entomol..

[21] Yu S.J. (2008). The Toxicology and Biochemistry of Insecticides.

[22] Ffrench-Constant R.H. (2014). Insecticide resistance comes of age. Genome Biol..

[23] Hodgson E., Kerkut G.A., Gilbert L.C. (1985). Microsomal monooxigenases. Comprehensive Insect Physiology, Biochemistry and Pharmacology.

[24] Gomori G. (1953). Human esterases. J. Lab. Clin. Med..

[25] Van Asperen K. (1962). A study of housefly esterases by means of a sensitive colorimetric method. J. Insect Physiol..

[26] Brogdon W.G., Dickinson C.M. (1983). A microassay system for measuring esterase activity and protein concentration in small samples and in high-pressure liquid chromatography eluate fractions. Anal. Biochem..

[27] Devonshire A.L., Moores G., Ffrench-Constant R. (1986). Detection of insecticide resistance by immunological estimation of carboxylesterase activity in Myzus persicae (Sulzer) and cross reaction of the antiserum with Phorodon humuli (Schrank) (Hemiptera: Aphididae). Bull. Entomol. Res..

[28] Dary O., Georghiou G.P., Parsons E., Pasteur N. (1990). Microplate adaptation of Gomori’s assay for quantitative de-termination of general esterase activity in single insects. J. Econ. Entomol..

[29] Van De Baan H.E., Croft B.A. (1990). Factors Influencing Insecticide Resistance in Psylla pyricola (Homoptera: Psyllidae) and Susceptibility in the Predator Deraeocoris brevis (Heteroptera: Miridae). Environ. Entomol..

[30] Van De Baan H.E., Croft B.A. (1991). Resistance to insecticides in winter and summer forms of pear psylla, Psylla pyricola. Pestic. Sci..

[31] Berrada B., Fournier D., Cuany A., Nguyen T.X. (1994). Identification of resistance mechanisms in a selected laboratory strain of Cacopsylla pyri (Homoptera: Psyllidae): Altered Acetylcholinesterase and detoxifying oxidases. Pestic. Biochem. Physiol..

[32] Sauphanor B., Cuany A., Bouvier J.C., Brosse V., Amichot M., Bergé J.B. (1997). Mechanism of resistance to deltamethrin in Cydia pomonella (L.) (Lepidoptera: Tortricidae). Pestic. Biochem. Physiol..

[33] Martin T., Chandre F., Ochou O., Vaissayre M., Fournier D. (2002). Pyrethroid resistance mechanisms in the cotton bollworm Helicoverpa armigera (Lepidoptera: Noctuidae) from West Africa. Pestic. Biochem. Physiol..

[34] Maymó A.C., Cervera A., Garcerá M.D., Bielza P., Martinez-Pardo R. (2006). Relationship between esterase activity and acrinatrin and methiocarb resistance in field populations of western flower thrips, Frankliniella occidentalis. Pestic Manag. Sci..

[35] Bouvier J.-C., Cuany A., Monier C., Brosse V., Sauphanor B. (1998). Enzymatic diagnosis of resistance to deltamethrin in diapausing larvae of the codling moth, Cydia pomonella (L.). Arch. Insect Biochem. Physiol..

[36] Habig W.H., Pabst M., Jakoby W.B. (1974). The first enzymatic step in mercapturinc acid formation. J. Biol. Chem..

[37] Habig W.H., Jakoby W.B. (1981). Assays for differentiation of glutathione S-Transferases. Methods Enzymol..

[38] Scott J.G. (1999). Cytochromes P450 and insecticide resistance. Insect Biochem. Mol. Biol..

[39] Civolani S., Cassanelli S., Rivi M., Manicardi G.C., Peretto R., Chicca M., Pasqualini E., Leis M. (2010). Survey of Susceptibility to Abamectin of Pear Psylla (Hemiptera: Psyllidae) in Northern Italy. J. Econ. Entomol..

[40] Feyereisen R. (1999). INSECT P450 ENZYMES. Annu. Rev. Entomol..

[41] Guengerich F.P., Martin M.V., Sohl C.D., Cheng Q. (2009). Measurement of cytochrome P450 and NADPH–cytochrome P450 reductase. Nat. Protoc..

[42] Yuan C.Y., Jing T.X., Li W., Liu X.Q., Liu T.Y., Liu Y., Chen M.L., Jiang R.X., Yuan G.R., Dou W. (2021). NADPH-cytochrome P450 reductase mediates the susceptibility of Asian citrus psyllid Diaphorina citri to imidacloprid and thiamethoxam. Pestic. Manag. Sci..

[43] Ortego F., López-Olguín J., Ruiz M., Castañera P. (1999). Effects of toxic and deterrent terpenoids on digestive protease and detoxication enzyme activities of Colorado potato beetle larvae. Pestic. Biochem. Physiol..

[44] Civolani S., Boselli M., Butturini A., Chicca M., Cassanelli S., Tommasini M.G., Aschonitis V., Fano E.A. (2015). Testing spirotetramat as an alternative solution to abamectin for Cacopsylla pyri (Hemiptera: Psyllidae) control: Laboratory and field Tests. J. Econ. Entomol..

[45] Comins H.N. (1977). The development of insecticide resistance in the presence of migration. J. Theor. Biol..

[46] Rodríguez M.A., Mârques T., Bosch D., Avilla J. (2011). Assessment of insecticide resistance in eggs and neonate larvae of Cydia pomonella (Lepidoptera: Tortricidae). Pestic. Biochem. Physiol..

[47] El Saidy M.F. (1991). Biological and Biochemical Activities of Benzoylphenylureas and Conventional Insecticides on Spodoptera littoralis. Ph.D. Thesis.

[48] Berrada S., Nguyen T.X., Merzoug D., Fournier D. (1995). Selection for monocrotophos resistance in pear psylla, Cacopsylla pyri(L.) (Hom., Psyllidae). J. Appl. Entomol..

[49] Buès R., Boudinhon L., Toubon J.F., d’Arcier F. (1999). Geographic and seasonal variability of resistance to insecticides in Cacopsylla pyri L. (Hom., Psyllidae). J. Appl. Entomol..

[50] Buès R., Bouvier J., Boudinhon L. (2005). Insecticide resistance and mechanisms of resistance to selected strains of Helicoverpa armigera (Lepidoptera: Noctuidae) in the south of France. Crop. Prot..

[51] Grant D.F., Bender D.M., Hammock B.D. (1989). Quantitative kinetic assays for glutathione S-transferase and general esterase in individual mosquitoes using an EIA reader. Insect Biochem. Mol. Biol..

[52] Masters B.S.S., Kamin H., Gibson Q.H., Williams C.H. (1965). Studies on the mechanism of microsomal triphosphopyridine nucleotide-cytochrome c reductase. J. Biol. Chem..

[53] Rogers A., Gibon Y., Schwender J. (2009). Enzyme Kinetics: Theory and Practice in Plant Metabolic Networks.

[54] Lehninger A.L. (1988). Principios de Bioquímica.

[55] Voet D., Voet J.G. (2006). Bioquímica.

[56] Smagghe G., Tirry L., Ishaaya I. (2001). Insect midgut as a site for insecticide detoxification and resistance. Biochemical Sites of Insecticide Action and Resistance.

[57] Margoliash E., Frohwirt N. (1959). Appendix—Spectrum of horse-heart cytochrome c. Biochem. J..

[58] Bradford M.M. (1976). A rapid and sensitive method for the quantification of microgram quantities of protein utilizing the principle of protein-dye binding. Anal. Biochem..

[59] Robertson J.L., Preisler H.K., Russell R.M. (2002). PoloPlus, Probit and Logit Analysis, User’s Guide.

[60] Esmaeily M., Talebi K., Hosseininaveh V., Nozari J. (2019). Seasonal variation in susceptibility of the pear psyllid, Cacopsylla permixta (Hemiptera: Psyllidae) to selected insecticides. J. Appl. Entomol..

[61] Feyereisen R., Baldridge G., Farnsworth D. (1985). A rapid method for preparing insect microsomes. Comp. Biochem. Physiol. Part B Comp. Biochem..

[62] Williams C.H., Kamin H. (1962). Microsomal Triphosphopyridine Nucleotide-Cytochrome c Reductase of Liver. J. Biol. Chem..

[63] Gacesa P., Hubble J. (1990). Tecnología de las Enzimas.

[64] Navarro-Roldán M., Bosch D., Gemeno C., Siegwart M. (2020). Enzymatic detoxification strategies for neurotoxic insecticides in adults of three tortricid pests. Bull. Entomol. Res..

[65] Reyes M., Barros-Parada W., Ramírez C.C., Fuentes-Contreras E. (2015). Organophosphate Resistance and its Main Mechanism in Populations of Codling Moth (Lepidoptera: Tortricidae) from Central Chile. J. Econ. Entomol..

[66] Rodríguez M.A., Bosch D., Sauphanor B., Avilla J. (2010). Susceptibility to organophosphate insecticides and activity of detoxifying enzymes in Spanish populations of Cydia pomonella (Lepidoptera: Tortricidae). J. Econ. Entomol..

